# Evaluation of registration accuracy using short‐arc cone‐beam computed tomography for image‐guided radiotherapy in breast cancer

**DOI:** 10.1002/acm2.70804

**Published:** 2026-10-06

**Authors:** Shuhei Wada, Masahide Tominaga, Motoharu Sasaki, Koichi Yamanashi, Yoshio Iwai, Shunsuke Furutani, Yusei Shinohara, Yoshihiro Uto

**Affiliations:** ^1^ Graduate School of Advanced Science and Technology Tokushima University Tokushima Tokushima Japan; ^2^ Division of Radiology Kansai Medical University Hospital Hirakata Osaka Japan; ^3^ Graduate School of Biomedical Sciences Tokushima University Tokushima Tokushima Japan; ^4^ Canon Medical Systems Corporation Otawara Tochigi Japan; ^5^ Research Physics Minato‐ku Tokyo Japan; ^6^ Department of Radiology Tokushima Municipal Hospital Tokushima Tokushima Japan; ^7^ Graduate School of Technology, Industrial and Social Sciences Tokushima University Tokushima Tokushima Japan

**Keywords:** cone‐beam computed tomography, image‐guided radiotherapy, short arc

## Abstract

**Background:**

Image‐guided radiotherapy (IGRT) using cone‐beam computed tomography (CBCT) enables highly accurate imaging registration; however, increased radiation exposure remains a concern. Recently, short‐arc CBCT (SA‐CBCT), which reduces projection data and reconstructs images, has become available. Although previous studies have shown that image quality deteriorates when projection data are reduced, no study has directly evaluated the clinical usefulness of SA‐CBCT or its effect on registration accuracy.

**Purpose:**

We verified the registration accuracy of SA‐CBCT in breast tangential irradiation using an anthropomorphic phantom and clinical data, and examined its clinical feasibility compared with the commonly used 200° scan.

**Methods:**

An anthropomorphic phantom was placed on the treatment bed, and CT and CBCT images acquired during treatment planning were aligned. Bed movement was measured for both conventional CBCT and SA‐CBCT images. The difference between the two measurements was quantified as *N*, defined as the norm of the vector difference between CBCT and SA‐CBCT registration shifts, to evaluate registration accuracy. The acquisition angle was varied to assess the effects of central and total angles. In the clinical study, registration data from 30 consecutive patients who underwent whole‐breast irradiation were analyzed. Data were reconstructed by changing the acquisition angle from 200° to 120°, and *N* was calculated as in the phantom experiment.

**Results:**

In the phantom experiment, *N* was within 0.15 mm (3‐axis) and 0.55 mm (6‐axis) when the acquisition angle was reduced to 120°. In clinical data, the average *N* was 0.64 mm in 3‐axis registration (84.7% within 1 mm) and 0.85 mm in 6‐axis registration (71.3% within 1 mm). Even with a 120° acquisition angle, the body contour and chest wall edges were sufficiently preserved.

**Conclusions:**

SA‐CBCT maintained adequate registration accuracy in breast tangential irradiation. These findings suggest its clinical applicability with appropriate acquisition angle settings.

## 1 INTRODUCTION

Cone‐beam computed tomography (CBCT) is used in many centers for image‐guided radiotherapy (IGRT). CBCT is useful for reducing interfractional errors because it enables more precise positioning by using the positional information about the patient's body immediately before treatment.^1^, ^2^ However, the increased radiation dose resulting from imaging registration is a concern. The American Association of Physicists in Medicine (AAPM) TG75^3^ and TG180^4^ reports provide an overview and dose control for CBCT in IGRT. According to the TG180 report, the recommended threshold for imaging dose to be considered in the planning process is 5% of the therapeutic target dose. Although the dose from kV‐CBCT is usually below this threshold, it should be reduced according to the ‘as low as reasonably achievable’ (ALARA) principle.^5^, ^6^ There are various approaches to reducing the CBCT dose.^7^ These include the use of filters such as copper and bowtie filters,^8^, ^9^ optimization of scan parameters,^10^, ^11^ and statistical reconstruction.^12^, ^13^ Lu et al. attempted to reduce the dose by reducing the projection data to equally spaced intervals.^14^ In this study, experiments using a phantom demonstrated that even using only one‐sixth of the number of projections for a 360° scan may not considerably affect registration accuracy. Another method that has been proposed is high‐speed CBCT scanning, in which the number of projections is reduced by increasing the gantry rotation speed.^15^ However, high‐speed CBCT scans produce large noise and artifacts in the CBCT images, which affect the accuracy of IGRT.^15^ In addition, some studies used deep convolutional neural networks (DCNN) to improve the quality of these images.^16^ In addition to these reduction methods, Short‐Arc CBCT (SA‐CBCT) has recently attracted attention. CBCT typically collects information from acquisition angles of 360° or 200°, whereas SA‐CBCT reconstructs images from a smaller number of angles. SA‐CBCT is expected to reduce the patient dose and on‐couch time by reducing the acquisition angle. If SA‐CBCT can shorten the scanning time, it may be possible to acquire images from more patients with a single breath‐hold during deep inspiration breath‐holds (DIBH)^17^.However, a decrease in image quality is expected. It is essential to determine whether the decrease in image quality resulting from the reduced acquisition angle falls within the clinically acceptable range. In most cases, imaging registration in tangential breast irradiation is based on information such as body contour and chest wall. Therefore, even if the SA‐CBCT image quality is degraded, information such as the body contour and chest wall can still be utilized in clinical practice. Although previous studies have noted that image quality deteriorates when CBCT projection data are reduced,^14^ no study has directly evaluated the clinical usefulness of SA‐CBCT or its impact on registration accuracy.

Herein, we evaluated the registration accuracy of SA‐CBCT in breast tangential irradiation through experiments using an anthropomorphic phantom and clinical data and examined the feasibility of its clinical use by comparing it with the commonly used 200° scan.

## 2 METHODS

### 2.1 Experiments with an anthropomorphic phantom

The overall experimental procedure is outlined below. A treatment plan was created using an anthropomorphic phantom (PBU‐60, Kyoto Kagaku). CBCT or SA‐CBCT imaging was performed on the treatment bed, and the registration accuracy was compared.

#### 2.1.1 Treatment planning

We simulated tangential irradiation of the left breast using an anthropomorphic phantom. Images were acquired using a treatment‐planning CT scanner (Aquilion Lightning, Canon Medical Systems). The CT imaging conditions were as follows: a conventional scan with a tube voltage of 120 kV, tube current of CT‐AEC, scan speed of 3 s/rotation, slice thickness of 2 mm, reconstruction function of FC13, reconstruction interval of 2 mm, and FOV of 500 mm. The isocenter was set at 5 mm–1 cm from the chest wall, and a treatment plan for tangential breast irradiation was developed and registered to the radiotherapy machine (Versa HD, Elekta).

#### 2.1.2 CBCT and SA‐CBCT imaging

For CBCT imaging, the phantom was set up on the treatment bed, and images were acquired. During the setup, considering that some misalignment would occur when an actual patient lays on the bed, the phantom was displaced approximately 5 mm from the isocenter set during treatment planning along the X‐ (lateral), Y‐ (longitudinal), and Z‐ (vertical) axes.

Figure 1a shows a schematic of the central and acquisition angles. CBCT images were acquired at central angle of 135° with a 200° angular acquisition range, respectively. The acquisition conditions were tube voltage of 100 kV, tube current of 20 mA/frame, and acquisition time of 10 ms/frame. The reconstruction function was S20‐Med_Res, and the voxels were resampled into 1 mm isotropic voxels. To evaluate the registration accuracy for different central and acquisition angles, we acquired SA‐CBCT images by changing the central and acquisition angles based on this image (CBCT image).

**FIGURE 1:**
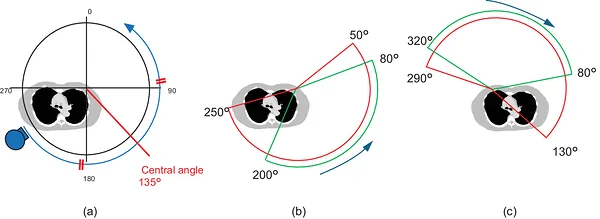
Scan geometry. (a) Schematic of the central angle, acquisition angle and acquisition area. The central angle is the angle of the center of the rotation range of the kV tube. The CBCT images were acquired from the projection data at a central angle of 135° and an acquisition angle of 200°. (b) and (c) Schematic of the acquisition area. The red area in the figure represents the acquisition angle of 200°, and the green area represents the acquisition angle of 120°. (b) Left breast (c) Right breast.

In SA‐CBCT image acquisition, the acquisition angle was fixed at 120°, which was considerably reduced, and the central angle was changed from 100° to 210° in 10° increments to evaluate the registration accuracy due to the difference in central angle. Next, to evaluate the registration accuracy for different acquisition angles, the central angle was fixed at 135°, the acquisition angle was decreased by 10° from 200° to 100°, and the images were captured.

#### 2.1.3 Imaging registration

Imaging registration was performed using the CT, CBCT, and SA‐CBCT images obtained from the treatment planning system. The registration range is shown in Figure 2. It was set to the area that included the midline and center of the body thickness in the horizontal cross‐section, and the craniocaudal direction was set to a range that fully included the irradiation range. Automatic registration was used for registration using GrayValue T and GrayValue T+R. GrayValue mode is an algorithm that performs automatic registration by matching the grayscale intensity values of voxels through cross‐correlation.^18^ GrayValue T is registration in the translation direction along the X‐, Y‐, and Z‐axes, whereas GrayValue T+R is registration in six axes that adds the rotation directions of ROLL, PITCH, and YAW to the translation direction.

**FIGURE 2:**
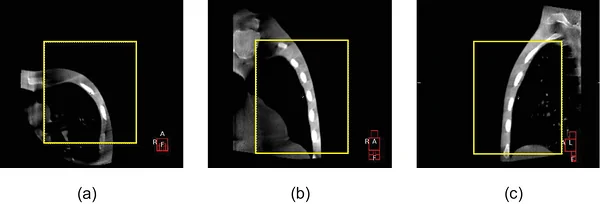
Registration range. (a) Transverse, (b) sagittal, (c) coronal.

#### 2.1.4 Evaluation method

The evaluation was based on the difference in the registration errors between CBCT and SA‐CBCT during automatic registration. $d_{cbct}$ and $d_{SA-cbct}$ were calculated for X, Y, Z, YAW, ROLL, and PITCH when the CBCT and SA‐CBCT images were matched automatically. From Equation (1), the difference value D was calculated for the X‐, Y‐, and Z‐axes, YAW, ROLL, and PITCH, respectively, and denoted $D_{x}$, $D_{y}$, $D_{z}$, $D_{YAW}$, $D_{ROLL}$, and $D_{PITCH}$. For the translation direction, norm N was calculated from $D_{x}$, $D_{y}$, and $D_{z}$ using Equation (2).

Table 1 presents the evaluation methods used for each registration method. In the case of registration using GrayValue T, evaluation was performed using the norm of the difference value N because only the translation direction was involved. In the case of registration using GrayValue T+R, the evaluation was performed using the norm of the difference value N for the translation direction, and the difference values $D_{YAW}$, $D_{ROLL}$, and $D_{PITCH}$ for the rotation direction. Here, the norm of the difference value N quantifies the registration discrepancy between SA‐CBCT and CBCT and does not represent the absolute error relative to the true value.

(1) $$\text{Difference value}\;D=d_{CBCT}-d_{SA-CBCT}$$

(2) $$\text{Norm of the difference value}\;N=\sqrt{D_{X}^{2}+D_{Y}^{2}+D_{Z}^{2}}$$

**TABLE 1:** Evaluation methods for each registration algorithm.

	GrayValue T	GrayValue T+R
Translational direction	Norm of the difference value N	Norm of the difference value N
Rotational direction		Difference value $D_{YAW}$, $D_{ROLL}$, $D_{PITCH}$

#### 2.1.5 Image analysis of SA‐CBCT

Using an image processing software, a Laplacian filter was applied to the CBCT images, and the SA‐CBCT images were collected at 120° to extract the edges. Next, the edges were compared and evaluated at the 40°, 45°, and 50° lines centered at the lower‐right corner of the matching area.

### 2.2 Experiments using clinical images

This retrospective study included 30 consecutive patients who underwent whole breast irradiation without nodal irradiation at our hospital. All procedures involving human subjects were conducted in accordance with relevant legal and institutional guidelines and were approved by our Hospital Ethics Committee (approval number R4‐10). Informed consent was obtained from all participants prior to their inclusion in the study, and their privacy rights were strictly observed.

We used CBCT images taken over five days, from the first to the fifth day of treatment, with an acquisition angle of 200°. These patients are 30 consecutive patients treated since January 2023. The participants were women aged 50 to 90 years, with a mean age of 68.7 years. We performed registration with the CBCT images based on the CT images used at the time of treatment planning and calculated the registration error $d_{cbct}$. Next, we reduced the acquisition angle and performed a reconstruction to create a pseudo‐SA‐CBCT image. Similar to the CBCT images, we performed registration and calculated the registration error, $d_{SA-cbct}$. Finally, we calculated the difference between $d_{cbct}$ and $d_{SA-cbct}$ and evaluated it.

#### 2.2.1 Creation of SA‐CBCT images

To evaluate registration accuracy at different acquisition angles, we performed reconstruction based on CBCT images collected at 200° by incrementing the acquisition angle by 10° from 120° to 200°. Reconstruction was performed under the same conditions described in Section 2.1.2.

Figure 1b shows a schematic diagram of the acquisition range of 200–120° for tangential irradiation of the left breast. For the left breast, reconstruction was performed with a central angle of 140° when the acquisition angle was 120°, a central angle of 145° when the acquisition angle was 130°, and a central angle of 150° when the angular range of acquisition was between 140° and 200°. Figure 1c shows a schematic diagram of the acquisition range of 200–120° for tangential irradiation of the right breast. For the right breast, reconstruction was performed with a central angle of 20° when the acquisition angle was 120°, a central angle of 25° when the acquisition angle was 130°, and a central angle of 30° when the angular range of acquisition was between 140° and 200°.

#### 2.2.2 Imaging registration

Imaging registration was performed within the same range as described in Section 2.1.3. However, considering the possibility that imaging registration could not be performed with only one registration, two registrations were performed, and the data from the second registration were used.

#### 2.2.3 Evaluation methods in experiments with clinical data

As described in Section 2.1.4, evaluation was performed based on the difference in registration error between CBCT and SA‐CBCT during automatic registration. $d_{cbct}$ and $d_{SA-cbct}$ were calculated for the X‐, Y‐, and Z‐axes. From Equation (1), the difference value D was calculated for the X‐, Y‐, and Z‐axes and designated $D_{x}$, $D_{y}$, and $D_{z}$, respectively. Norm N was calculated from $D_{x}$, $D_{y}$, and $D_{z}$ using Equation (2).

#### 2.2.4 Comparison of CBCT (200° acquisition) and SA‐CBCT (120° acquisition)

The SA‐CBCT images were reconstructed at an acquisition angle of 120°, and the norm N between the SA‐CBCT and CBCT images was calculated and evaluated. Subsequently, for 11 cases with 3‐axis registration and 30 cases with 6‐axis registration among patients with a difference exceeding 1.2 mm, SA‐CBCT images were created by expanding the imaging angle from 120° to 190°. The norm of the difference value N was calculated for each acquisition angle, and the registration accuracy was evaluated.

### 2.3 Dose measurement using an ionization chamber for CBCT and SA‐CBCT

An ionization chamber (DC300, IBA Dosimetry) connected to an electrometer (RAMTEC Solo, Toyo Medic) was placed at the isocenter. Measurements were performed in air without the anthropomorphic phantom. CBCT imaging was performed with a 200° acquisition angle, and SA‐CBCT imaging was performed with acquisition angles from 190° to 120°. The imaging conditions were the same as those described in Section 2.1.2. At each acquisition angle, the ionization measurement was repeated five times, and the mean value was used. The ratio of the central dose in SA‐CBCT imaging to that in CBCT imaging at a 200° acquisition angle was calculated.

### 2.4 Comparison of acquired SA‐CBCT and reconstructed SA‐CBCT

To confirm that simulated SA‐CBCT images reconstructed from 200° projection data were equivalent to actually acquired SA‐CBCT images, a phantom validation experiment was performed. Using the anthropomorphic phantom, CBCT images were acquired at a 200° acquisition angle, and SA‐CBCT images were acquired at acquisition angles from 180° to 120°. Simulated SA‐CBCT images with acquisition angles from 180° to 120° were reconstructed by reducing the projection data from the 200° acquisition. The difference value D between the acquired and reconstructed SA‐CBCT images was calculated.

## 3 RESULTS

### 3.1 Experimental results on the anthropomorphic phantom

#### 3.1.1 Difference by central angle

Figure 3 shows examples of SA‐CBCT images at different central angles. As shown by the red circles in Figure 3, artifacts appeared when the central angles were 160°, 150°, 120°, and 110°, and the body contour and chest wall information necessary for registration were missing. In contrast, when the central angles were 140° and 130°, the artifacts were reduced, and the body contour and chest wall information were sufficiently preserved. Figure 5a shows the registration results obtained using the GrayValue T. When the central angle was in the range of 130° to 170°, the norm of the difference value N was within 0.15 mm on average, confirming stable registration accuracy. In the case of GrayValue T+R (Figure 5b), the central angle at which the difference in the translation direction was the smallest was 130° to 150°. However, in the rotational direction (Figure 5c), the difference was within 0.2° when the central angle was in the range of 130–210°. From this result, it is considered that a central angle of 130–140° is optimal.

**FIGURE 3:**
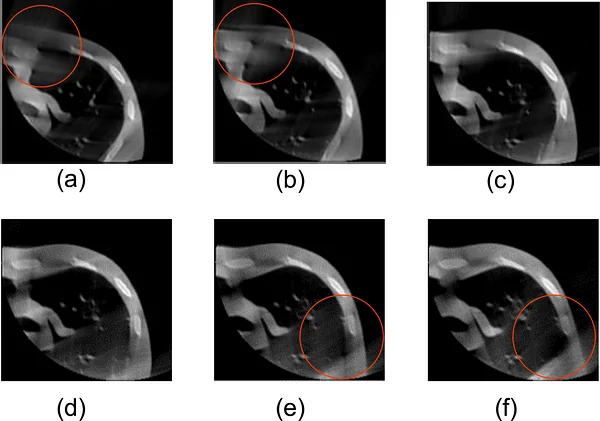
SA‐CBCT images. All acquisition angles were set to 120°. The central angles were (a) 160°, (b) 150°, (c) 140°, (d) 130°, (e) 120°, and (f) 110°.

#### 3.1.2 Difference by acquisition angle

Figure 4 shows an example of SA‐CBCT images acquired at different acquisition angles. The quality of the SA‐CBCT images worsened as the acquisition angle decreased. Figure 5d shows the result of registration using GrayValue T. Even when the acquisition angle was reduced to 120°, the norm of the difference value N was within 0.15 mm on average, and sufficient registration accuracy was obtained. Here, when the acquisition angle was 100°, the difference value N was 9.95 mm, which was significantly larger than values for other acquisition angles. This is thought to be due to missing information about the body contour and chest wall, as can be seen in Figure 4f. In the case of GrayValue T+R (Figure 5e), the norm N was within 0.55 mm at all acquisition angles in the translation direction. In addition, in the rotational direction (Figure 5f), the difference was within 0.2° at all acquisition angles, except when the acquisition angle was 100°. These results indicate that registration accuracy can be sufficiently guaranteed even when the acquisition angle is approximately 120°.

**FIGURE 4:**
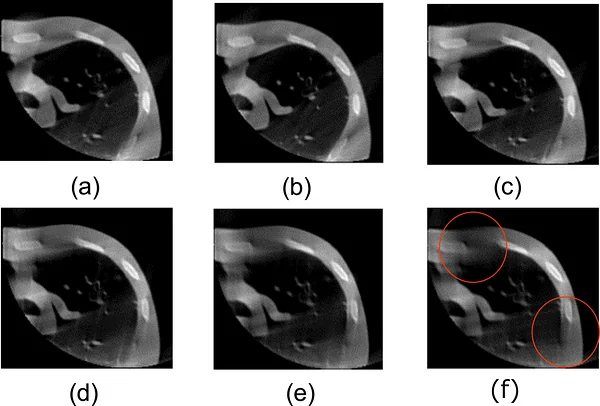
SA‐CBCT images. The central angle was 135°. The acquisition angles were (a) 150°, (b) 140°, (c) 130°, (d) 120°, (e) 110°, and (f) 100°, respectively. In (f), the area circled in red is blurred in black and lacks information regarding the body contours and chest wall.

**FIGURE 5:**
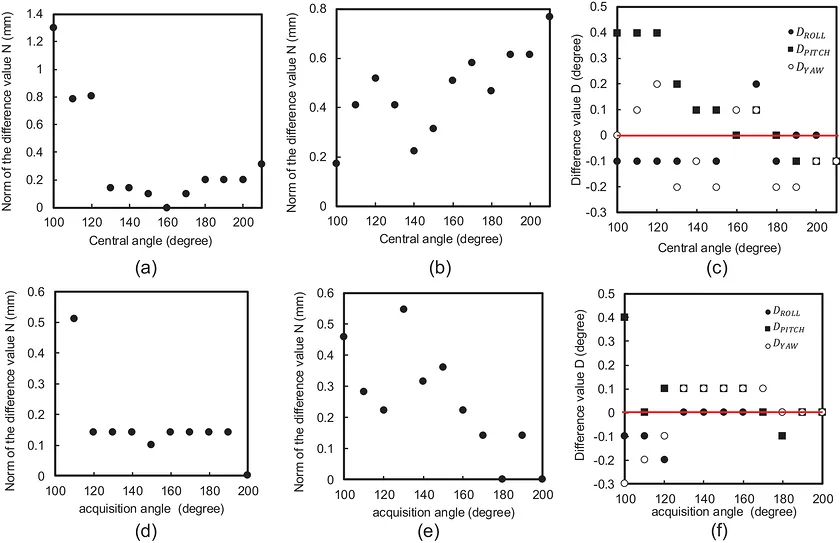
Phantom registration results. (a), (b) and (c) Norms of the difference value N at each central angle and the difference value D in the rotational direction. (a) Image registration with GrayValue T, (b) translational direction during registration with GrayValue T+R, and (c) rotational direction during registration with GrayValue T+R. (d), (e) and (f) Norms of the difference value N at each acquisition angle and the difference value D in the rotational direction. (d) Image registration with GrayValue T, (e) translational direction during registration with GrayValue T+R, and (f) rotational direction during registration with GrayValue T+R. In (d), the norm was 9.95 at an acquisition angle of 100°.

#### 3.1.3 Image analysis of SA‐CBCT images by edge extraction

Figure 6a shows the locations for comparison. Figure 6b,c, and d show of an edge comparison between CBCT and SA‐CBCT (acquisition angle of 120°). In most cases, the edges of the body contours and chest wall were preserved. In addition, there were differences in the profiles of locations other than the body contours and chest wall.

**FIGURE 6:**
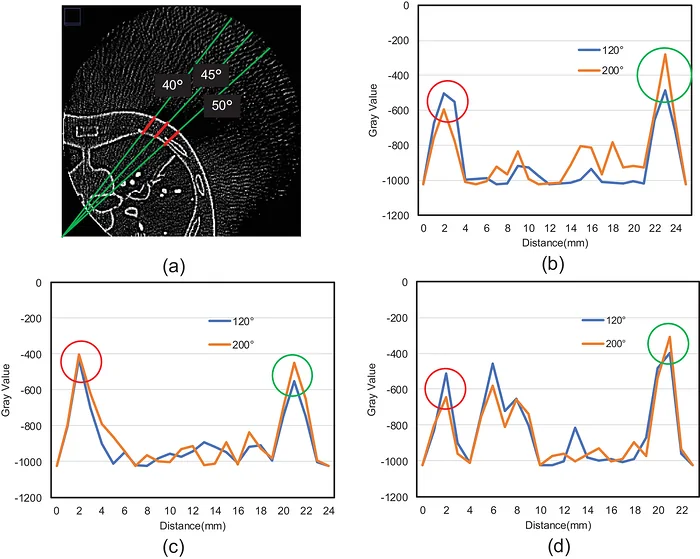
Comparison of profile curves of CBCT and SA‐CBCT images. (a) shows the locations for comparison. (b), (c), and (d) shows comparisons at the 40°, 45°, and 50° lines, respectively. The green circles in the figure indicate the body contours, and the red circles indicate the data at the coordinates of the chest wall.

### 3.2 Experimental results on clinical data

#### 3.2.1 Differences in SA‐CBCT images depending on the acquisition angle

As in the experiment with the anthropomorphic phantom, the image quality of the edges deteriorated as the acquisition angle decreased.

#### 3.2.2 Comparison of CBCT and SA‐CBCT (120°)

Figure 7a and b show the relationship between norm N and the number of cases. As shown in Figure 7a, when registration was performed using GrayValue T, the average norm N was 0.64 mm (95% CI, 0.57–0.72 mm) when the acquisition angle was reduced to 120°. The maximum norm N was 1.75 mm, and 84.7% of the total cases achieved a norm N of 1 mm or less. As seen from Figure 7b, when registration was performed using GrayValue T+R, the average norm N was 0.85 mm (95% CI, 0.67–1.0 mm) when the acquisition angle was reduced to 120°. The maximum norm N was 3.72 mm, and 71.3% of the total cases achieved a norm N of 1 mm or less.

**FIGURE 7:**
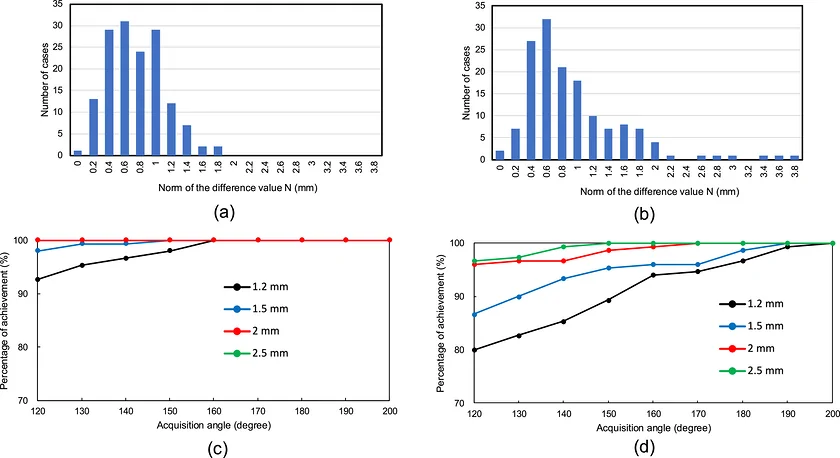
Clinical registration results. (a) and (b) Relationship between norm N and number of cases comparing CBCTs collected at 200° and SA‐CBCTs collected at 120°. (a) registration using GrayValue T and (b) registration using GrayValue T + R. (c) and (d) Acquisition angles and percentage of achievement for each norm N. The percentage of achievement was the percentage of norm N at the acquisition angle that met the criteria of 1.2, 1.5, 2, and 2.5 mm for all the images. (c) Registration using GrayValue T and (d) registration using GrayValue T+R.

Figure 7c and d show the relationship between the percentages of achievement for each norm N at different acquisition angles. Table 2 shows the acquisition angles achieved in all cases for each norm N. From Figure 7c, when registration was performed with GrayValue T, the rate at which a norm N of 1.2 mm was achieved was 92.7%, the rate at which a norm N of 1.5 mm was achieved was 98%, and the rate at which a norm N of 2 mm was achieved was 100% when the acquisition angle was set to 120°. From Figure 7d, when registration was performed with GrayValue T+R, the rate at which a norm N of 1.2 mm was achieved was 80%, the rate at which a norm N of 1.5 mm was achieved was 87%, the rate at which a norm N of 2 mm was achieved was 96%, and the rate at which a norm N of 2.5 mm was achieved was 97% when the acquisition angle was set to 120°.

**TABLE 2:** Acquisition angles achieved for all cases.

	1.2 mm	1.5 mm	2 mm	2.5 mm
GrayValue T	160°	150°	120°	120°
GrayValue T+R	200°	190°	170°	150°

### 3.3 Dose ratio between SA‐CBCT (120°) and CBCT (200°)

Figure 8 shows the ratio of central dose in SA‐CBCT imaging to that in CBCT imaging. The coefficient of determination of the regression curve was $R^{2}$= 0.99, indicating that the central dose was highly dependent on the acquisition angle. The central dose decreased as the acquisition angle decreased. When the acquisition angle was reduced to 120°, the central dose decreased by 33.4%.

**FIGURE 8:**
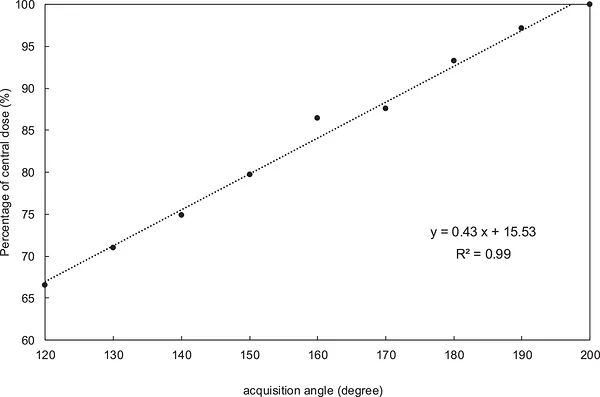
Ratio of central dose in SA‐CBCT imaging to CBCT imaging. The ratio of ionization amount at each acquisition angle was calculated relative to the ionization amount at an acquisition angle of 200°.

### 3.4 Comparison of acquired and reconstructed SA‐CBCT images

For acquisition angles of 130°–180°, the difference value was within 0.1 mm for both 3‐axis and 6‐axis matching. At a 120° acquisition angle, the maximum difference was 0.3 mm. These results suggest that the reconstructed simulated SA‐CBCT images were comparable to the actually acquired SA‐CBCT images.

## 4 DISCUSSION

This is the first study to quantitatively evaluate the effects of the acquisition and central angles of SA‐CBCT on registration accuracy and examine the possibility of its clinical application. In an experiment using an anthropomorphic phantom, the registration accuracy of SA‐CBCT was evaluated by varying the central and acquisition angles. As shown in Figure 3, when the central angle was varied, the SA‐CBCT image with an acquisition angle of 120° showed a prominent artifact on the ventral upper edge at a central angle of 150° and on the lateral side of the body at a central angle of 110°. However, the artifacts had little effect on the area around the breast at 130° or 140° from the center. This suggests that artifacts generated by the same acquisition angle vary depending on the central angle, and that these artifacts can be reduced by using an appropriate central angle. SA‐CBCT is conceptually related to digital breast tomosynthesis (DBT), because both use limited‐angle projection acquisition and may therefore exhibit limited‐angle artifacts^19^. However, DBT is intended for diagnostic visualization, whereas SA‐CBCT was used for IGRT localization in the present study. Thus, the clinical acceptability of SA‐CBCT should be assessed primarily by preservation of registration‐relevant anatomy, such as the body contour and chest wall, and by registration accuracy rather than diagnostic image quality.

In the evaluation based on the difference value, GrayValue T achieved stable registration accuracy when the central angle was 130–170°. For GrayValue T+R, the difference value was smallest when the central angle was 130–150°. The difference value in the translational direction differed between GrayValue T and Gray Value T+R. This is thought to be because GrayValue T+R includes correction in the rotational direction, which complicates the amount of movement for each of the X‐, Y‐, and Z‐axes.

When the acquisition angle was decreased, the image quality also decreased. This indicates that the image reconstruction was insufficient, owing to a decrease in the projection data. In GrayValue T+R, the norm of the difference value N was within 0.55 mm for all acquisition angles, although the norm N varied with the acquisition angle in the translational direction. In the rotational direction, the difference values D was good up to 110°. When the acquisition angle was 130°, the norm N in the translational direction was larger than that at the other acquisition angles. This is because the projection data decreased as the acquisition angle decreased, as shown in Figure 5e, where norm N gradually increased with decreasing acquisition angle up to 130°. In contrast, norm N decreased when the acquisition angle was 120°. This is because the difference, D, in the rotation direction was within 0.1° when the acquisition angle was reduced from 190° to 130°, whereas it was as large as 0.2° at 120°, as shown in Figure 5f. Based on the evaluation of the differences for various acquisition angles, it is concluded that automatic matching can be performed with high accuracy, even when the acquisition angle is reduced to 120°.

A comparison of the edges of the CBCT and SA‐CBCT images showed that the information on the body contour and chest wall was maintained even when the acquisition angle was reduced to 120°. In addition, there were differences in profiles other than the body contour and chest wall. This suggests that differences in the grey values of the edges may affect the matching accuracy.

In the experiment using clinical data, image quality decreased as the acquisition angle decreased, a finding consistent with that in an experiment using an anthropomorphic phantom. The achievement rates shown in Figure 7c and d generally increased with increasing acquisition angle, consistent with the trend observed in the phantom experiment. In whole breast irradiation, reported CTV‐PTV/setup margins are generally a few millimeters. In studies of whole breast irradiation using IGRT, margins of approximately 5 mm or more are often used or recommended^20^. Therefore, the results of 3‐axis and 6‐axis registration at a 120° acquisition angle are smaller than typical setup margins, suggesting that SA‐CBCT is clinically viable. As shown in Figure 7c and d, the percentage of each norm N that achieved each standard length was calculated. The results of the evaluation of these difference quantities confirmed the high accuracy of the registration in automatic matching, even when the acquisition angle decreased, as shown in the clinical data.

Measurements of the central dose showed that the dose decreased as the acquisition angle decreased. Therefore, a 33.4% dose reduction can be expected with a 120° SA‐CBCT. Furthermore, the acquisition time was approximately 33 s with an acquisition angle of 200° but was approximately 20 s with an acquisition angle of 120°. Based on these results, if the acquisition angle is set to 120°, it may be possible to perform imaging with a single breath hold during DIBH.

A limitation of this study lies in the fact that the clinical data experiments were artificially generated by reconstructing images that had already been taken. However, as shown in Result 3.4, there is no great difference between the SA‐CBCT images actually acquired and the simulated SA‐CBCT images obtained through reconstruction.

Another limitation is that noise was not quantified using the standard deviation in uniform ROIs. In SA‐CBCT, missing projection information can create regions with values of −1024, making such measurements primarily reflect reconstruction artifacts rather than stochastic noise. Therefore, we focused on edge preservation and registration accuracy, which are directly relevant to IGRT localization in tangential breast irradiation.

In addition, this study focused on breast tangential irradiation, and the findings may not be generalizable to areas dominated by soft tissue, such as the prostate or the head and neck. Furthermore, this study evaluated the alignment using only automatic matching. In actual clinical settings, final alignment is generally performed visually. Therefore, we believe that SA‐CBCT can be used in clinical settings.

We found that SA‐CBCT provides the necessary information for breast tangential irradiation positioning even when the acquisition angle is reduced to 120°, based on a comprehensive evaluation of the SA‐CBCT image and the calculated difference. Reducing the acquisition angle to 120° is expected to shorten the imaging time, reduce patient exposure dose, and decrease treatment time, including for IGRT. However, as the setup margin and required matching accuracy differ at each facility, it is recommended that the acquisition angle of SA‐CBCT be set with reference to the results of this study.

## 5 CONCLUSIONS

We evaluated the registration accuracy of SA‐CBCT for tangential breast irradiation. The results suggest that SA‐CBCT may be clinically applicable to tangential irradiation of the breast by setting the appropriate acquisition angle for SA‐CBCT, considering the setup margin and matching method.

## AUTHOR CONTRIBUTIONS

Shuhei Wada, Masahide Tominaga and Koichi Yamanashi designed the study, the main conceptual ideas, and the proof outline. Shuhei Wada, Masahide Tominaga and Shunsuke Furutani collected the data. Motoharu Sasaki, Yoshio Iwai, Yusei Shinohara and Yoshihiro Uto aided in interpreting the results and worked on the manuscript. Masahide Tominaga and Yoshihiro Uto supervised the project. Shuhei Wada wrote the manuscript with support from Masahide Tominaga and Yoshihiro Uto. All authors discussed the results and commented on the manuscript.

## CONFLICT OF INTEREST STATEMENT

Conflict of interest statement Koichi Yamanashi is an employee of Canon Medical, and Yoshio Iwai is an employee of Elekta

## ETHICS STATEMENT

This study was conducted in accordance with the Declaration of Helsinki and was approved by the Ethics Committee of Tokushima Municipal Hospital (Approval No.: R4‐10).

## Data Availability

Due to the nature of this research, participants of this study did not agree for their data to be shared publicly, so supporting data is not available.
